# Association of exposure level to passive smoking with hypertension among lifetime nonsmokers in Japan: a cross-sectional study

**DOI:** 10.1097/MD.0000000000013241

**Published:** 2018-11-30

**Authors:** Takashi Tamura, Yuka Kadomatsu, Mineko Tsukamoto, Rieko Okada, Tae Sasakabe, Sayo Kawai, Asahi Hishida, Megumi Hara, Keitaro Tanaka, Ippei Shimoshikiryo, Toshiro Takezaki, Isao Watanabe, Daisuke Matsui, Takeshi Nishiyama, Sadao Suzuki, Kaori Endoh, Kiyonori Kuriki, Yoshikuni Kita, Sakurako Katsuura-Kamano, Kokichi Arisawa, Hiroaki Ikezaki, Norihiro Furusyo, Yuriko N. Koyanagi, Isao Oze, Yohko Nakamura, Haruo Mikami, Mariko Naito, Kenji Wakai

**Affiliations:** a Department of Preventive Medicine, Nagoya University Graduate School of Medicine, Nagoya; b Department of Public Health, Aichi Medical University School of Medicine, Nagakute; c Department of Preventive Medicine, Faculty of Medicine, Saga University, Saga; d Department of International Island and Community Medicine, Kagoshima University Graduate School of Medical and Dental Sciences, Kagoshima; e Department of Epidemiology for Community Health and Medicine, Kyoto Prefectural University of Medicine, Kyoto; f Department of Public Health, Nagoya City University Graduate School of Medical Sciences, Nagoya; g Laboratory of Public Health, Division of Nutritional Sciences, School of Food and Nutritional Sciences, University of Shizuoka, Shizuoka; h Faculty of Nursing Science, Tsuruga Nursing University, Tsuruga; i Department of Preventive Medicine, Institute of Biomedical Sciences, Tokushima University Graduate School, Tokushima; j Department of Environmental Medicine and Infectious Disease, Graduate School of Medical Sciences, Kyushu University, Fukuoka; k Division of Cancer Information and Control, Aichi Cancer Center Research Institute, Nagoya; l Division of Cancer Epidemiology and Prevention, Aichi Cancer Center Research Institute, Nagoya; m Cancer Prevention Center, Chiba Cancer Center Research Institute, Chiba; n Department of Oral Epidemiology, Hiroshima University Graduate School of Biomedical and Health Sciences, Hiroshima, Japan.

**Keywords:** blood pressure, exposure to passive smoking, hypertension, Japan, nonsmokers

## Abstract

Brief exposure to passive smoking immediately elevates blood pressure. However, little is known about the association between exposure to passive smoking and chronic hypertension. We aimed to examine this association in a cross-sectional study, after controlling multiple potential confounders.

Participants included 32,098 lifetime nonsmokers (7,216 men and 24,882 women) enrolled in the Japan Multi-Institutional Collaborative Cohort Study. Passive smoking was assessed using a self-administered questionnaire. The single question about exposure to passive smoking had five response options: “sometimes or almost never,” “almost every day, 2 hours/day or less,” “almost every day, 2 to 4 hours/day,” “almost every day, 4 to 6 hours/day,” and “almost every day, 6 hours/day or longer.” Hypertension was defined as any of the following: systolic blood pressure ≥140 mmHg, diastolic blood pressure ≥90 mmHg, or use of antihypertensive medication. Multivariate-adjusted odds ratio (OR) and 95% confidence interval (CI) for hypertension were estimated by exposure level to passive smoking using unconditional logistic regression models.

The multivariate-adjusted OR for hypertension in those exposed almost every day was 1.11 (95% CI: 1.03–1.20) compared with those exposed sometimes or almost never. The OR for a 1-hour per day increase in exposure was 1.03 (95% CI: 1.01–1.06, *P*_*for trend*_ = .006). This association was stronger in men than in women; the ORs were 1.08 (95% CI: 1.01–1.15, *P*_*for trend*_ = .036) and 1.03 (95% CI: 1.00–1.05, *P*_*for trend*_ = .055), respectively.

Our findings suggest importance of tobacco smoke control for preventing hypertension.

## 1 Introduction

Hypertension is a strong risk factor for cardiovascular disease, which is considered one of the greatest public health concerns worldwide.^[1,2]^ Some previous studies have demonstrated that exposure to passive smoking elevates blood pressure for a short period, up to 24 hours.^[3–5]^ This can be partially explained by several biological effects caused by exposure to passive smoking, such as vasoconstriction mediated by nicotine-induced catecholamine release, endothelial dysfunction, and decreased nitric oxide production.^[5–8]^ Growing evidence has shown that passive smoking is associated with morbidity and mortality from cardiovascular diseases,^[9–11]^ suggesting that individuals should avoid exposure to passive smoking to prevent cardiovascular disease. These findings imply that daily exposure to passive smoking might be associated with the incidence of hypertension. To the best of our knowledge, however, little is known about the association between exposure to passive smoking and hypertension among lifetime nonsmokers. To date, we know of seven epidemiological studies that have succeeded in showing positive associations between exposure to passive smoking and elevated blood pressure in general adult populations;^[12–18]^ however, the generalizability of those results was limited by small sample sizes and/or restricted regions. Therefore, it can be expected that examining the association between passive smoking and hypertension among nonsmokers in a large general population would yield better understanding of this association, which could help health authorities in decision-making about policies for health promotion and intervention to prevent hypertension.

The present study aimed to examine whether the exposure level to passive smoking is associated with the prevalence of hypertension among nonsmokers, in a cross-sectional analysis with adjustment for multiple confounders, using data from a large cohort study in Japan.

## 2 Methods

### 2.1 Participants

Participants in the present study were enrolled in the Japan Multi-Institutional Collaborative Cohort (J-MICC) Study.^[19]^ The J-MICC Study is a large cohort study in Japan, launched in 2005, to identify interactions between genetic and lifestyle factors for lifestyle-related diseases, including any cancer. The details and rationale of the J-MICC Study have been described elsewhere.^[20]^ Briefly, participants completed a self-administered questionnaire about lifestyle and medical information and donated a peripheral blood sample during a health check-up at the baseline survey. The J-MICC Study included residents in the community, health check examinees, and patients of a cancer hospital. All participants in the study provided their written informed consent. The study protocol was approved by the Ethics Committees of Nagoya University Graduate School of Medicine, Aichi Cancer Center, and other institutions participating in the J-MICC Study.

A total of 92,642 participants were recruited from 14 different areas throughout Japan between 2004 and 2013 (the dataset used in the present study was fixed on January 11, 2018). The study areas included Chiba, Shizuoka-Sakuragaoka, Okazaki, Aichi, Shizuoka, Daiko, Iga, Takashima, Kyoto, Tokushima, Saga, Kagoshima, Fukuoka and the Kyusyu and Okinawa Population Study (KOPS). Of the total participants, we excluded 37,103 from four areas (Chiba, Aichi, Fukuoka, and KOPS) from this study because there were no available data on exposure to passive smoking (Fukuoka and KOPS) or measured blood pressure (Chiba and Aichi). To examine the effect of exposure to passive smoking on blood pressure by excluding the effect of active smoking, we limited participants to those with nearly no experience of active smoking in their lifetime, leaving 33,293 nonsmokers (i.e., those who had smoked less than 365 cigarettes during their lifetime). We further excluded those with missing data for exposure to passive smoking, blood pressure, and use of antihypertensive medication (n = 182, 990 and 23, respectively). Thus, the present study included 32,098 nonsmokers (7,216 men and 24,882 women) in the final analysis.

### 2.2 Assessment of blood pressure, exposure level to passive smoking, and lifestyle factors

We used a self-administered questionnaire that included the following demographic characteristics and lifestyle factors: alcohol consumption, smoking status, exposure to passive smoking, education level, physical activity, psychological stress, sleeping hours, family and personal medical history, current medication, and menstruation in women.

Height and weight were directly measured on the day of the survey; body mass index (BMI) was calculated as weight in kilograms divided by the square of height in meters (kg/m^2^). Each participant's blood pressure was measured by a nurse or trained staff in the health check-up, using a standard mercury sphygmomanometer or an automated blood pressure measurement monitor, with the patient in a seated position. Exposure level to passive smoking was assessed using a single question: “Have you inhaled the smoke of cigarettes from others (i.e., smokers) at home, in an office, or elsewhere in the last year?” with the following five response options: “sometimes or almost never,” “almost every day, 2 hours/day or less,” “almost every day, 2 to 4 hours/day,” “almost every day, 4 to 6 hours/day,” and “almost every day, 6 hours/day or longer.” For the present analysis, men who answered “almost every day, 4 to 6 hours/day” and “almost every day, 6 hours/day or longer” were combined into the group “almost every day, 4 hours/day or longer” because of the small number of men who responded “almost every day, 6 hours/day or longer” (n = 55). Physical activity was estimated as metabolic equivalent hours per day according to the frequency and duration of daily and leisure time activities.^[21,22]^ Ethanol intake (g/day) was estimated for current drinkers (defined as those who consumed alcohol at least once a week during the last year), based on the reported consumption frequency and amount consumed per one time for six alcoholic beverages (i.e., Japanese sake, shochu, shochu-based cocktails, beer, whisky, and wine).^[23]^

### 2.3 Statistical analyses

Participant characteristics were presented as mean ± standard deviation (SD) for continuous variables and as number and proportion for categorical variables in each level of exposure to passive smoking. The differences in the means or proportions between exposure levels to passive smoking were tested using analysis of variance or the chi-squared test, respectively.

Hypertension was defined according to any of the following criteria: systolic blood pressure ≥140 mmHg, diastolic blood pressure ≥90 mmHg, or use of antihypertensive medication.^[24,25]^ Crude, age- and sex-adjusted, and multivariate-adjusted odds ratios (ORs) and 95% confidence intervals (CIs) for hypertension were estimated by exposure level to passive smoking using unconditional logistic regression models. The multivariate-adjusted model included the following covariates: age (as a continuous variable), sex (for men and women combined only), alcohol consumption (never, former, current drinkers who consumed <23 g/day ethanol, current drinkers who consumed ≥23 g/day ethanol), education level (≤9, 10–15, ≥16 years), BMI (<18.5, 18.5 to <25, ≥25 kg/m^2^), physical activity (as a continuous variable), psychological stress (not at all, not much, a little, a lot), sleeping hours (<6, 6 to <8, ≥8 hours), family history of hypertension (yes, no for both father and mother), medical history of diabetes mellitus (yes, no), medical history of dyslipidemia (yes, no), menstruation (premenopausal, perimenopausal, postmenopausal for women only) and study area (Shizuoka-Sakuragaoka, Okazaki, Shizuoka, Daiko, Iga, Takashima, Kyoto, Tokushima, Saga, Kagoshima). Participants with missing data for covariates were included as additional categories in the analysis. The linear trend for risk was evaluated using weight proportional to the exposure level to passive smoking (as a continuous variable for 1 hour). The assigned scores were as follows: 0 for “sometimes or almost never,” 1 for “almost every day, 2 hours/day or less,” 3 for “almost every day, 2 to 4 hours/day,” 5 for “almost every day, 4 to 6 hours/day,” and 6 for “almost every day, 6 hours/day or longer.”

A *P* value of <.05 was considered statistically significant. All statistical analyses were performed using SAS 9.4M5, which runs on SAS University Edition (SAS Institute Inc., Cary, NC).

## 3 Results

Table 1 presents characteristics of 32,098 study participants according to exposure levels to passive smoking. Mean age ± SD was 54.9 ± 9.5 years, and the proportion of men was 22.5%. Current drinkers accounted for 71.4% of men and 37.1% of women. Participants with higher exposure levels to passive smoking were significantly more likely to be young, women, less educated, obese, physically active, stressed, and to have fewer sleeping hours. The distributions of medical history of dyslipidemia and study area were also significantly different among exposure levels to passive smoking. There were no significant differences in alcohol consumption, family history of hypertension, and medical history of diabetes mellitus among the exposure levels.

**Table 1:**
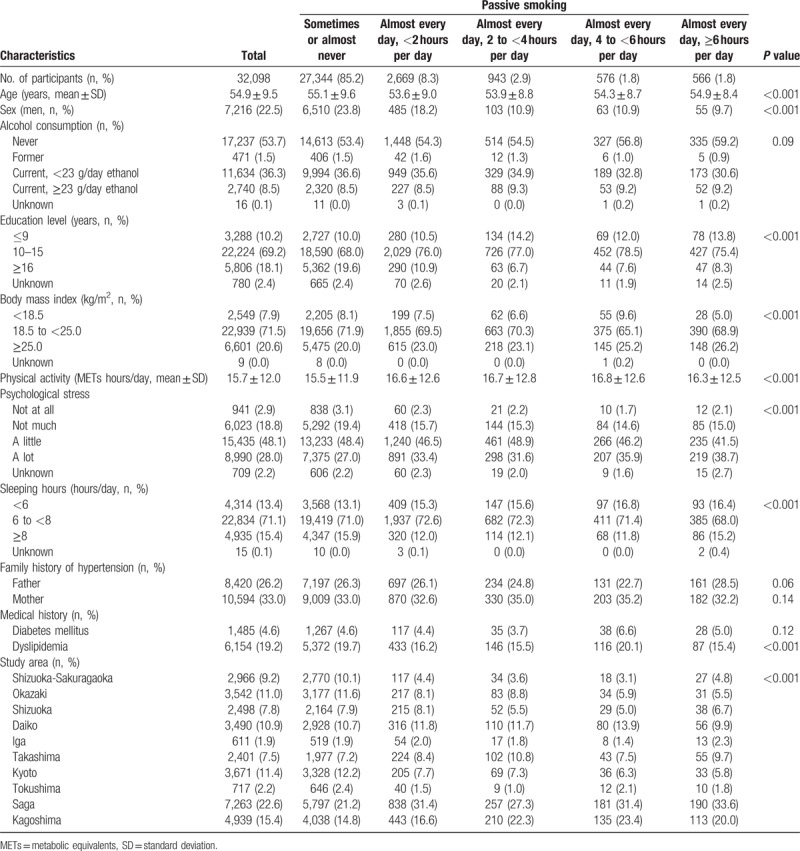
Participant characteristics according to exposure level to passive smoking among nonsmokers.

The multivariate-adjusted ORs and 95% CIs for hypertension according to the presence of exposure to passive smoking are shown in Table 2. A total of 10,100 participants (2,814 men and 7,286 women, representing 31.5% of the participants analyzed) had hypertension. Individuals exposed to passive smoking almost every day had a significantly higher risk of hypertension compared with those exposed sometimes or almost never; the multivariate-adjusted OR was 1.11 (95% CI: 1.03–1.20). In the analysis stratified by sex, the positive association was significant only for men, although the direction of association was not different by sex; the OR was 1.21 (95% CI: 1.00–1.46) for men and 1.09 (95% CI: 0.99–1.18) for women.

**Table 2:**
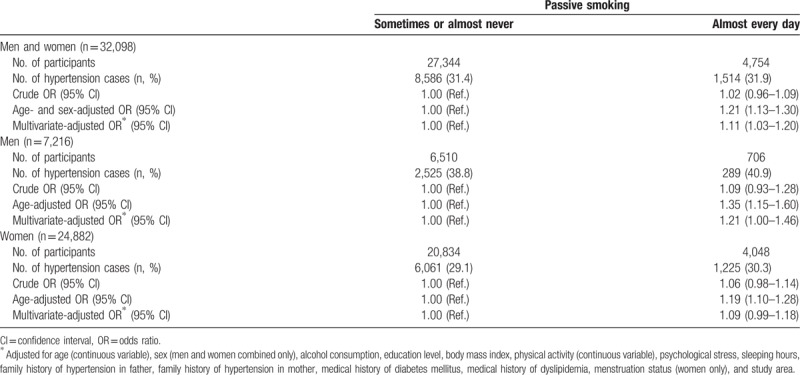
ORs and 95% CIs for hypertension according to exposure to passive smoking among nonsmokers.

Table 3 shows the association between exposure levels to passive smoking and risk of hypertension. The risk significantly increased with exposure level to passive smoking; the multivariate-adjusted OR for a 1-hour per day increase in exposure was 1.03 (95% CI: 1.01–1.06, *P*_*for trend*_ = .006). In particular, those exposed almost every day, 4 to <6 hours per day, had a significantly increased risk of hypertension; the multivariate-adjusted OR was 1.26 (95% CI: 1.04–1.54) compared with participants exposed sometimes or almost never. When the analysis was stratified by sex, this association persisted and appeared to be stronger in men than in women; the OR for a 1-hour per day increase in exposure was 1.08 (95% CI: 1.01–1.15, *P*_*for trend*_ = .036) in men and 1.03 (95% CI: 1.00–1.05, *P*_*for trend*_ = .055) in women.

**Table 3:**
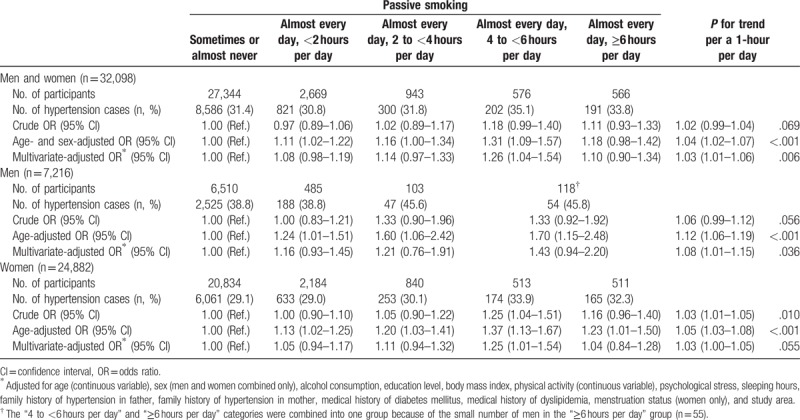
ORs and 95% CIs for hypertension according to exposure level to passive smoking among nonsmokers.

## 4 Discussion

In this large cross-sectional study from Japan, the presence of exposure to passive smoking was found to be significantly positively associated with the prevalence of hypertension among nonsmokers, independently of multiple potential confounders, including medical history. The OR for hypertension increased according to exposure level to passive smoking with a significant linear trend, and the association was stronger in men than in women.

Studies exploring the effect of passive smoking on hypertension have been scarce. To the best of our knowledge, seven epidemiological studies in general adult populations have demonstrated a positive association with a small sample size or in restricted regions.^[12–18]^ A previous cross-sectional study in Japan showed that those exposed to daily passive smoking had increased systolic blood pressure by approximately 4 mmHg in the morning;^[13]^ however, it remains unclear whether exposure to passive smoking is associated with the prevalence of hypertension. We revealed that individuals exposed to passive smoking almost every day have an 11% greater risk of hypertension compared with those exposed only sometimes or almost never, after controlling for multiple confounders. In addition, the OR for hypertension increased according to exposure level to passive smoking with a significant linear trend; the increased risk for a 1-hour per day increase in exposure was 8% in men and 3% in women. Only one study has examined the association between serum cotinine levels and hypertension among never smokers in an adult population in the United States; cotinine in the body is the principal metabolite of nicotine contained mainly in cigarettes and is considered a biomarker for exposure to smoking.^[14]^ In that study, the result for the highest quartile of serum cotinine level (≥0.218 ng/mL) was consistent with that in our male participants who were exposed almost every day, 4 hours per day or longer; the multivariate-adjusted OR for hypertension was 1.44 (95% CI: 1.01–2.04) compared with the lowest quartile of serum cotinine level (≤0.025 ng/mL). Regarding women, a previous cross-sectional study in China examining the association between the husband's smoking and wife's hypertension showed findings consistent with those for women in the present study; the multivariate-adjusted OR for hypertension in women was 1.28 (95% CI: 1.27–1.30) for the group in which only the husband smoked compared with the group in which both the husband and wife were nonsmokers.^[16]^ A most recent study in Korea also has shown that nonsmoking women exposed to passive smoking every day, 2 hours per day or longer showed a higher prevalence of hypertension; the multivariate-adjusted OR was 1.50 (95% CI: 1.10–2.04) compared with those without the exposure.^[18]^ Our findings reinforce the previously reported evidence for the association between exposure to passive smoking and hypertension.

It is well known that both active and passive smoking immediately elevate blood pressure for a short period in experimental settings.^[3–6,26]^ The link between smoking and elevated blood pressure appears to be biologically plausible because nicotine acts as an adrenergic agonist, inducing local or systemic catecholamine release at the neuroeffector junction or the release of vasopressin^[6]^; however, even findings regarding the chronic effects of active smoking on blood pressure have been inconclusive.^[26,27]^ In fact, several cross-sectional studies have failed to provide any evidence for or have demonstrated a negative association between smoking and blood pressure.^[28–31]^ Several studies, however, have shown positive associations.^[32–34]^ In addition, longitudinal studies examining the risk score for predicting incidence of hypertension have shown that smoking is significantly associated with onset of hypertension after multivariate adjustment^[35–37]^ whereas other studies have shown no association.^[38,39]^ The reasons for this inconsistency remain unclear. Nicotine-induced vasoconstriction is responsible for an acute transient increase in blood pressure,^[6]^ and this state is followed by decreased blood pressure as a consequence of nicotine's depressant effects. In the long term, carbon monoxide directly plays a role in causing endothelial dysfunction of the arterial wall^[8]^ and structurally irreversible alterations, such as arterial stiffness.^[40]^ These changes caused by active and passive smoking could potentially be associated with a state of chronically increased blood pressure or hypertension.^[41]^ Further studies are required to confirm the association of passive smoking with hypertension in the general population.

The present study was conducted with a large national sample and controlling for multiple potential confounders, adding to the strengths of our study. One study limitation was that data on exposure level to passive smoking were based on a self-administered questionnaire; thus, misclassification might have resulted from our evaluation of passive smoking. However, if present, this would have been nondifferential and led to underestimation of the associations. Some previous studies also have demonstrated that self-reported exposure to passive smoking among nonsmokers could be useful in epidemiological studies,^[42–44]^ and the self-administered questionnaire is the most commonly used tool to ascertain exposure to passive smoking.^[45]^

In the present study, the positive association between passive smoking and hypertension was stronger in men than in women; the reasons for this remain unclear. The difference in the strength of the association according to sex stems from the fact that the strength of the exposure might differ by sex, even in the same response category of exposure time to passive smoking, for reasons such as occupation or other confounders.^[44,46]^ We could not further clarify this issue owing to the lack of occupation data. It might also be necessary to consider the genetic background of participants regarding blood pressure, as approximately 31% to 68% of blood pressure variation could be genetically explained.^[47]^ However, genetic background appeared to be unrelated to exposure to passive smoking because we observed no clear difference in family history of hypertension between the exposure levels. Lastly, the cross-sectional nature of the study does not permit us to draw definite conclusions regarding the causal role of exposure to passive smoking in hypertension among nonsmokers.

In conclusion, exposure to passive smoking was found to be associated with the prevalence of hypertension in a large general Japanese population, with a significant linear trend after controlling for potential confounders. A government survey on public health and nutrition in Japan, 2017, has reported that 7.4% of adults were exposed to passive smoking every day at home and 30.1% of adults were exposed at least once a month in offices.^[48]^ As two-thirds of all deaths attributable to passive smoking were caused by ischemic heart disease,^[49]^ it is possible that hypertension related to passive smoking contributes to the death of ischemic heart disease due to passive smoking. Thus, a total ban on smoking at public venues and further efforts to raise public awareness about the dangers of passive smoking to hypertension and related diseases would be important to reduce the risk for these diseases. Our findings may add evidence to the recommendation for greater restrictions on tobacco smoke in public venues as well as at home or in offices, and emphasize the importance of intervention strategies for tobacco smoke control in preventing hypertension.

## Author contributions

**Conceptualization:** Takashi Tamura.

**Data curation:** Takashi Tamura, Yuka Kadomatsu, Mineko Tsukamoto, Rieko Okada, Tae Sasakabe, Sayo Kawai, Asahi Hishida, Mariko Naito, Kenji Wakai.

**Formal analysis:** Takashi Tamura.

**Funding acquisition:** Mariko Naito, Kenji Wakai.

**Investigation:** Takashi Tamura, Yuka Kadomatsu, Mineko Tsukamoto, Rieko Okada, Tae Sasakabe, Sayo Kawai, Asahi Hishida, Megumi Hara, Keitaro Tanaka, Ippei Shimoshikiryo, Toshiro Takezaki, Isao Watanabe, Daisuke Matsui, Takeshi Nishiyama, Sadao Suzuki, Kaori Endoh, Kiyonori Kuriki, Yoshikuni Kita, Sakurako Katsuura-Kamano, Kokichi Arisawa, Hiroaki Ikezaki, Norihiro Furusyo, Yuriko N. Koyanagi, Isao Oze, Yohko Nakamura, Haruo Mikami, Mariko Naito, Kenji Wakai, the Japan Multi-Institutional Collaborative Cohort (J-MICC) Study.

**Project administration:** Mariko Naito.

**Supervision:** Kenji Wakai.

**Visualization:** Takashi Tamura.

**Writing – original draft:** Takashi Tamura, Kenji Wakai.

**Writing – review & editing:** Takashi Tamura, Toshiro Takezaki, Kenji Wakai.

Takashi Tamura orcid: 0000-0002-1057-744X.
